# In Situ Prior Proliferation of CD4^+^ CCR6^+^ Regulatory T Cells Facilitated by TGF-β Secreting DCs Is Crucial for Their Enrichment and Suppression in Tumor Immunity

**DOI:** 10.1371/journal.pone.0020282

**Published:** 2011-05-31

**Authors:** Lin Xu, Wei Xu, Zhenke Wen, Sidong Xiong

**Affiliations:** 1 Institutes of Biology and Medical Sciences, Soochow University, Suzhou, China; 2 Institute for Immunobiology and Department of Immunology, Shanghai Medical College of Fudan University, Shanghai, China; 3 Department of Immunology, Zunyi Medical College, Zunyi, Guizhou Province, China; University of Sheffield, United Kingdom

## Abstract

**Background:**

CD4^+^CD25^+^ regulatory T cells (Tregs), a heterogeneous population, were enrichment in tumor mass and played an important role in modulating anti-tumor immunity. Recently, we reported a Treg subset, CCR6^+^ Tregs but not CCR6^−^Tregs, were enriched in tumor mass and closely related to poor prognosis of breast cancer patients. However, the underlying mechanism remains elusive. Here, we carefully evaluate the enrichment of CCR6^+^Tregs in tumor mass during progression of breast cancer and explore its possible mechanism.

**Methodology/Principal Findings:**

The frequency of CCR6^+^Tregs in tumor infiltrating lymphocytes (TILs ) was analyzed at early stage and at late stage of tumor in a murine breast cancer model by FACS respectively. The expansion of CCR6^+^Tregs and their CCR6^−^ counterpart in tumor mass were determined by BrdU incorporation assay. The effect and its possible mechanism of tumor-resident antigen presenting cells (APCs) on the proliferation of CCR6^+^Tregs also were evaluated. The role of local expansion of CCR6^+^Tregs in their enrichment and suppression in vivo also was evaluated in adoptive cell transfer assay. We found that the prior enrichment of CCR6^+^Tregs but not CCR6^−^Tregs in tumor mass during progression of murine breast cancer, which was dependent on the dominant proliferation of CCR6^+^ Tregs in situ. Further study demonstrated that tumor-resident DCs triggered the proliferation of CCR6^+^Treg cells in TGF-β dependent manner. Adoptive transfer of CCR6^+^Tregs was found to potently inhibit the function of CD8^+^T cells in vivo, which was dependent on their proliferation and subsequently enrichment in tummor mass.

**Conclusions/Significance:**

Our finding suggested that CCR6^+^ Tregs, a distinct subset of Tregs, exert its predominant suppressive role in tumor immunity through prior in situ expansion, which might ultimately provide helpful thoughts for the designing of Treg-based immunotherapy for tumor in the future.

## Introduction

CD4^+^CD25^+^ regulatory T cells (Treg), a subpopulation of CD4^+^ T cells constitutively expressing transcription factor forkhead box protein3 (Foxp3), consist 5–10% peripheral CD4^+^T cells in normal human and mice [1]. CD4^+^CD25^+^ Tregs effectively suppress the proliferation and activity of both CD4^+^CD25^−^ and CD8^+^ T cells in a contact-dependent manner through inhibition of interleukin 2 (IL-2) production [2]. Accumulating data have indicated that Tregs were enriched in tumor mass and potently inhibited the anti-tumor immunity mediated by CD4^+^Th1 and CD8^+^CTL [3], [4]. However, the exact mechanism of Tregs were enriched in tumor mass remains not fully understood.

Recently, some findings have indicated that there are distinct subsets of Tregs which play different roles in diverse animal models, mediating immune suppression or immune tolerance [5]–[9]. However, whether a distinct subset of Tregs is present in tumor environment and their role in mediating immunosuppression remains to be elucidated [10]. Previous study demonstrated a new subset of Tregs, which express CC chemokine receptor type 6 (CCR6), played an important role in the pathogenesis of experimental allergic encephalomyelitis (EAE) [11]. Kitamura et al further found that CCR6^+^ regulatory T cells contributed to the pathogenesis of experimental colitis [12]. In the setting of tumors, Lamprecht et al reported that CCR6^+^Tregs might favor immune escape of Hodgkin/Reed-Sternberg (HRS) cells [13]. Our recent work further showed that CCR6^+^ subset of Treg cells were dominantly enriched in tumor mass and closely related to poor prognosis of breast cancer patients [14]. Combing these data suggested that CCR6^+^Tregs might play a critical role in immunosuppression of anti-tumor immunity. However, the underlying mechanism of the enrichment of this Treg subset in tumor mass remains to be elucidated, which might be helpful for the understanding of mechanisms of contribution of distinct Treg subsets to immunosuppression and ultimately aid the designing of therapy for treating tumor patients.

To this end, in the present study, the distribution of CCR6^+^ Tregs was evaluated in a murine breast cancer model. Our data showed that CCR6^+^Tregs were dominantly enriched in the tumor mass during tumor progression. Notably, we provided evidence that the predominant proliferation of CCR6^+^ Tregs was crucial for their enrichment and suppressive effects *in vivo*. Finally, the possible role of tumor resident DCs in the proliferation of CCR6^+^ Tregs was studied.

## Results

### CCR6^+^Foxp3^+^ regulatory T cells were enriched in tumor mass during progression of breast tumor

**Figure 1:**
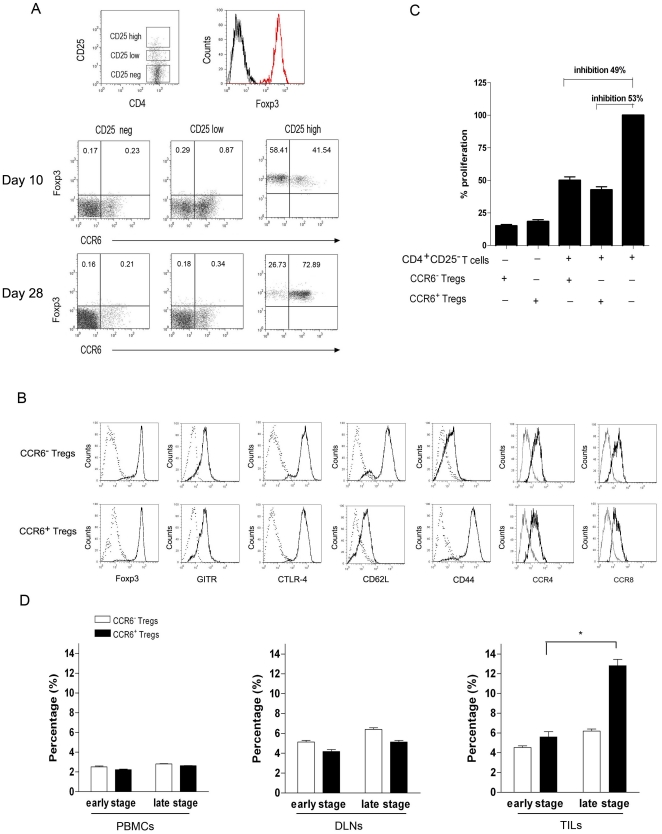
CCR6^**+**^Foxp3^**+**^ regulatory T cells were enriched in tumor mass during tumor progress. (**A**) Tumor infiltrating cells (TILs) from 4T1 bearing mice on day 10 or day 28 were stained by anti-CD4, anti-CD25, anti-Foxp3 and anti-CCR6 antibodies and analyzed by Flow cytometry. The gating strategy and expression of Foxp3 were shown. Dot plots showing examples of CCR6/Foxp3 expression in CD4^+^ CD25^neg^, CD4^+^ CD25^low^, and CD4^+^ CD25^high^ gated populations of TILs from 4T1 bearing mice are reported. (**B**) FACS analysis of the phenotypes of CCR6^−^ regulatory T cells and CCR6^+^ regulatory T cells. Dot lines here represent the isotype control. (**C**) Suppressive capacity of the sorted CCR6^−^ regulatory T cells and CCR6^+^ regulatory T cells against CD4^+^ CD25^−^Teff cells, determined by [^3^H]-thymidine incorporation respectively. (**D**) As gated on fig. 1a, the percentage of CCR6^−^ Foxp3^+^ regulatory T cells and CCR6^+^ Foxp3^+^ regulatory T cells in CD4^+^ T cells from peripheral blood mononuclear cells (PBMCs), draining lymph nodes(DLNs) and TILs were analyzed at day 10 or day 28 (termed as early or late stage) (n = 12) by FACS and calculated. *p<0.05.

Our previous data found that CCR6^+^Foxp3^+^ regulatory T cells were dominantly enriched in tumor mass and closely related to poor prognosis of breast cancer patients [14], to further investigate the mechanism of the enrichment of this tumor-resident Treg subset, the frequency of CCR6^+^ Tregs in TILs during tumor progression was observed. As shown in fig. 1a, the expression of CCR6 and Foxp3 were evaluated inside the CD4^+^ CD25^neg^, CD4^+^ CD25^low^ and CD4^+^ CD25^high^ gated populations of TILs from 4T1 bearing mice. We found that CCR6 was preferentially expressed on CD4^+^ CD25^high^ Foxp3^+^ regulatory T cells and defined a distinct subset of CCR6^+^CD4^+^ CD25^high^ Foxp3^+^ regulatory T cells in 4T1 bearing mice (Fig. 1a). Next, we analyzed the phenotype and inhibitory function of CCR6^+^Tregs. The expression level of Foxp3, GITR, CTLA-4, CCR4 and CCR8 on CCR6^+^ Tregs were similar to those on CCR6^−^ Tregs indicating both CCR6^+^ and CCR6^−^ Tregs displayed regulatory phenotype (Fig. 1b). And the inhibitory effect of CCR6^+^Tregs on responder CD4^+^ CD25^−^T cells was comparable to that of CCR6^−^ Tregs (Fig. 1c, p>0.05). Of note, CCR6^+^ Tregs expressed low level of CD62L and high level of CD44, displaying an effector/memory phenotype (Fig. 1b). We further analyzed the frequency of CCR6^+^Tregs and CCR6^−^Tregs at day 10 or 28 (defined as early and late stage respectively) and found that the CCR6^−^Treg frequency in TILs (6.1%) at late stage of tumor was equal to that (5.9%) at early stage of tumor; while the frequency of CCR6^+^Tregs in TILs (12.5%) at late stage of tumor was significantly higher than that (5.7%) at early stage of tumor (Fig. 1d, p<0.05). Moreover, CCR6^+^Treg frequency at late stage of tumor in TILs was higher than that in DLNs; In contrast, frequency of CCR6^−^ Tregs in TILs at late stage was similar to that in DLNs (Fig. 1d, p>0.05). In addition, there wasn't any change in the frequency of CCR6^+^Tregs or CCR6^−^Tregs in PBMCs during tumor progression. These data showed that CCR6^+^ Tregs, but not their CCR6^−^ counterpart, accumulated in tumor mass during tumor progression.

### Expansion of CCR6^+^Tregs contributed to their accumulation

**Figure 2:**
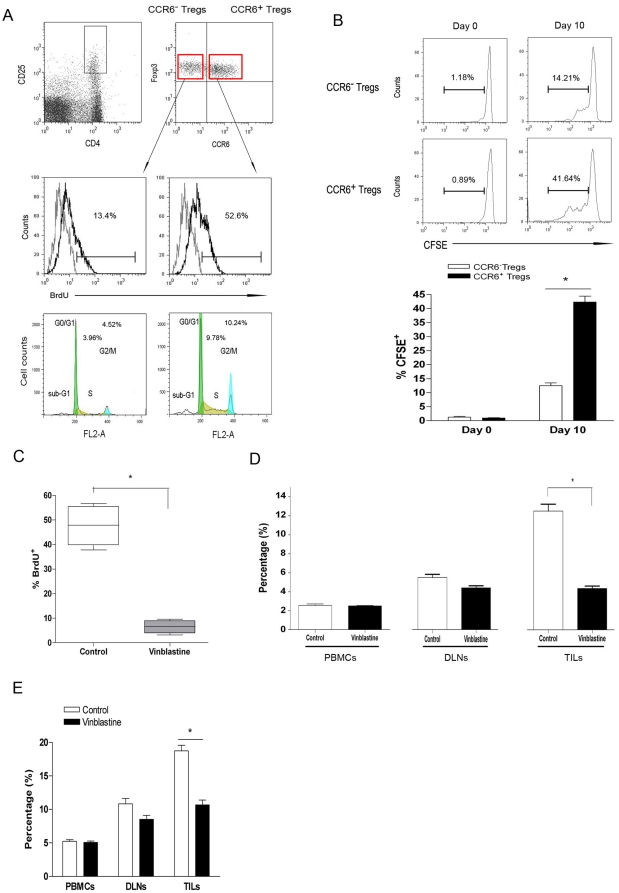
In situ expansion of CCR6^+^Tregs contributed to their prior accumulation in tumor mass. (**A**) The 4T1-bearing mice were treated with 2 mg BrdU i.p. every other day up to a cumulative dose of 8 mg BrdU. Then TILs was collected and analyzed. The gating strategy was shown. The proliferation and cell cycle of CCR6^+^Tregs and CCR6^−^Tregs were analyzed by FACS. (**B**) CCR6^+^ Tregs and CCR6^−^Tregs were purified by FACS from TILs and labeled with CFSE respectivley. Then, 1×10^6^ CFSE-labeled cells were injected into tumor mass in 4T1-bearing mice. 10 days later, the proliferation of CFSE labeled CCR6^+^Tregs or CCR6^−^Tregs was analyzed by FACS. (**C**). 4T1 bearing Balb/c mice were injected with or without 200 ug vinblastine sulfate through tail vein as reported previously [15]. 15 hours later, CCR6^+^Tregs were sorted by FACS from TILs and the proliferation were analyzed by FACS and calculated. And the percentage of CCR6^+^Tregs in PBMCs, DLNs and TILs also were analyzed by FACS and calculated (**D**). (E) The percentage of CD4^+^CD25^high^ Tregs in PBMCs, DLNs and TILs also were analyzed by FACS and calculated. One representative data of three independent experiments was shown.* p<0.05.

We next tried to elucidate whether selective enrichment of CCR6^+^ Tregs in the tumor mass was related to their preferable recruitment or their prior in site proliferation. And it was found that CCR6^+^Tregs expressed equal level of CCR4 and CCR8 as that of CCR6^−^Tregs (Fig. 1b) and displayed simlar response to CCL17 and CCL22 to CCR6^−^Tregs did (Fig S1). In addition, the concentration of CCL17, CCL20 and CCL22 in supernatant also were determined (Fig S2). However, cultured supernatant of 4T1 tumor cells attracted similar counts of CCR6^+^ and CCR6^−^Tregs (Fig S3). It is seemingly recuitment of CCR6^+^Tregs didn't be mostly responsible for their dominate enrichment in tumor sites. Previous finding showed that Tregs could proliferate in tumor mass. Then, we presumed that the proliferation of CCR6^+^Tregs may differ from that of CCR6^−^Tregs, which led to the dominant enrichment of CCR6^+^Tregs. It was found that 52.6% CCR6^+^Tregs were BrdU-positive, significantly higher than 13.4% BrdU-positive- CCR6^−^Tregs (Fig. 2a, p<0.05) in tumor mass. Moreover, the percentage of S phage of CCR6^+^Tregs was also higher than that of CCR6^−^Tregs (p<0.05). And the proliferation of tumor infiltrating CCR6^+^Tregs was higher than that in DLNs and PBMCs (data not shown). To further confirm this observation, CCR6^+^ or CCR6^−^Tregs were sorted from 4T1 bearing mice and labeled with CFSE, then injected into tumor mass in 4T1 bearing syngeneic mice respectively. 10 days later, the proliferation of CCR6^+^Treg cells or CCR6^−^Treg cells was analyzed. It was found that the percentage of CFSE^+^ CCR6^+^Treg cells (41.64%) were significantly higher than that of CFSE^+^ CCR6^−^Treg cells (14.21%) (Fig. 2b, p<0.05) indicating that CCR6^+^Tregs could more powerfully proliferate than CCR6^−^Tregs in tumor mass.

To elucidate whether local prior proliferation of CCR6^+^Tregs contributed to their accumulation in tumor mass, we treated 4T1 bearing mice with vinblastine (a cell cycle inhibitor) as described previously [16]. As expected, vinblastine treatment significantly reduced the in situ proliferation of CCR6^+^Tregs (Fig. 2c, p<0.05) compared with control group. Importantly, the proportion of CCR6^+^Tregs in tumor mass in vinblastine treated groups also decreased significantly compared with control group (Fig. 2d, p<0.05). In contrast, there was little effect of vinblastine treatment on the proportion of CCR6^−^Tregs in tumor mass. Subsequently, vinblastine treatment also significantly reduced the proportion of Tregs in tumor mass (Fig. 2e, p<0.05). Of note, CCR4 and CCR8 expression on CCR6^+^ or CCR6^−^Tregs was not altered by vinblastine (data not shown) and CCL17 or CCL22 could equivalently chemotract vinblastine pretreated CCR6^+^ and CCR6^−^Tregs in vitro (unpublished data). Combining these data suggested that compared to CCR6^−^Tregs, local prior proliferation of CCR6^+^ Tregs was critical for their dominant accumulation in tumor mass.

### Tumor-resident DCs selectively triggered the proliferation of CCR6^+^Treg

**Figure 3:**
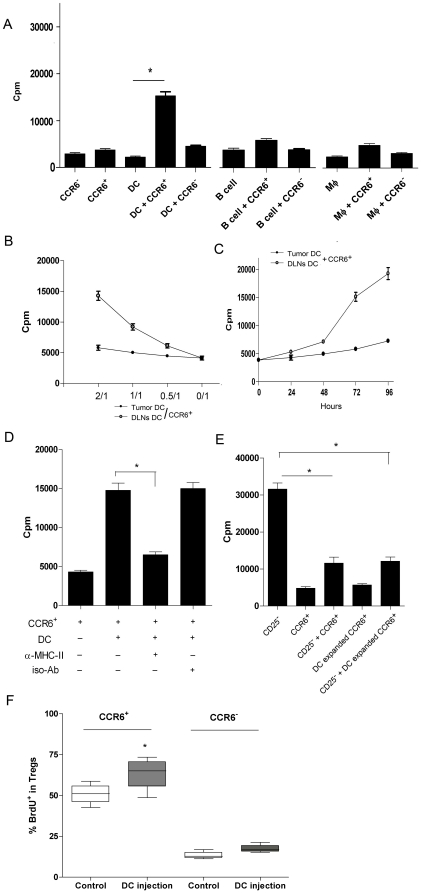
Tumor-resident DCs promoted the proliferation of CCR6^+^Tregs in situ. (**A**) CD11c^+^ DCs, macrophage or B cells were purified from TILs by MACS. 2×10^5^ CCR6^+^Tregs or CCR6^−^Tregs were cultured with CD11c^+^ DCs, macrophage or B cells from 4T1 bearing mice at a 1∶2 ratio respectively. 3 days later, the proliferation were determined by ^[3H]^thymidine incorporation assay. CD11c^+^ DCs were purified from TILs and DLNs by MACS. Proliferation of CCR6^+^Tregs after culturing with DCs from TILs or DLNs at indicated ratio was determined (**B**). (**C**) Proliferation of CCR6^+^Tregs after culturing with DCs from TILs or DLNs at a ratio of 1∶2 was determined at indicated time point as above described. (**D**) Proliferation of CCR6^+^Tregs after culturing with DCs at a ratio of 1∶2 in the presence of anti-MHC class II antibody (10 ug/ml) for 3days. (**E**) The inhibitory activity of CCR6^+^Tregs or DC-expanded CCR6^+^Tregs was determined. (**F**) 4×10^5^ CD11c^+^ DCs were purified from TILs and intratumorally injected into 4T1 syngeniec bearing mice (n = 8). 10 days later, the proliferation of CCR6^+^Tregs and CCR6^−^Tregs were analyzed by BrdU incorporation assay respectively as above described. One representative out of three independent experiments was shown. * p<0.05.

Since the local proliferation was crucial for the in vivo accumulation of CCR6^+^Tregs. Next we tried to find whether tumor-resident APCs could promote the proliferation of CCR6^+^ Tregs. Tumor-resident CD11c^+^ DCs, macrophages or B cells were isolated from tumor mass of 4T1 bearing mice and then cultured with CCR6^+^Tregs or CCR6^−^Tregs at 2∶1 ratio *in vitro*. It was shown that tumor-resident DCs significantly stimulated the proliferation of CCR6^+^ Tregs *in vitro* in a time and dose dependent manner (Fig. 3a,b and c, p<0.05), while no such effect was observed for any of the other APCs isolated from tumor mass (Fig. 3a, p>0.05). Moreover, there were moderate effect of tumor-resident DCs on CCR6^−^Tregs (Fig. 3a, p>0.05). In addition, DCs derived form DLNs had little effect on the proliferation of CCR6^+^Tregs (Fig. 3b,c), partially explaining why lower proliferation of CCR6^+^Tregs in DLNs was found than in TILs. Moreover, the DC-expanded CCR6^+^Tregs sustained their expression of Foxp3 and CCR6 (data not shown) and the suppressive function (Fig. 3e). Moreover, DCs induced the proliferation of CCR6^+^Treg in MHC-class II-dependent way (Fig. 3d, p<0.05). To further confirm the effect of DCs on the proliferation of CCR6^+^Tregs, we further intratumoral injected DCs into tumor mass in 4T1 bearing syngeneic mice and found that DCs could also significantly promote the proliferation of CCR6^+^Tregs in tumor mass (Fig. 3e, p<0.05). Similar to above data, we also find little effect of DCs on the proliferation of CCR6^−^Tregs in vivo (Fig. 3e, p>0.05). Different from the report that tumor cells could induce the proliferation of Tregs [17], we couldn't observe any effect of 4T1 tumor cells on the proliferation of CCR6^+^Tregs in vitro (data not shown).

#### DC-derived TGF-β was crucial for in situ expansion of CCR6^+^Treg

**Figure 4:**
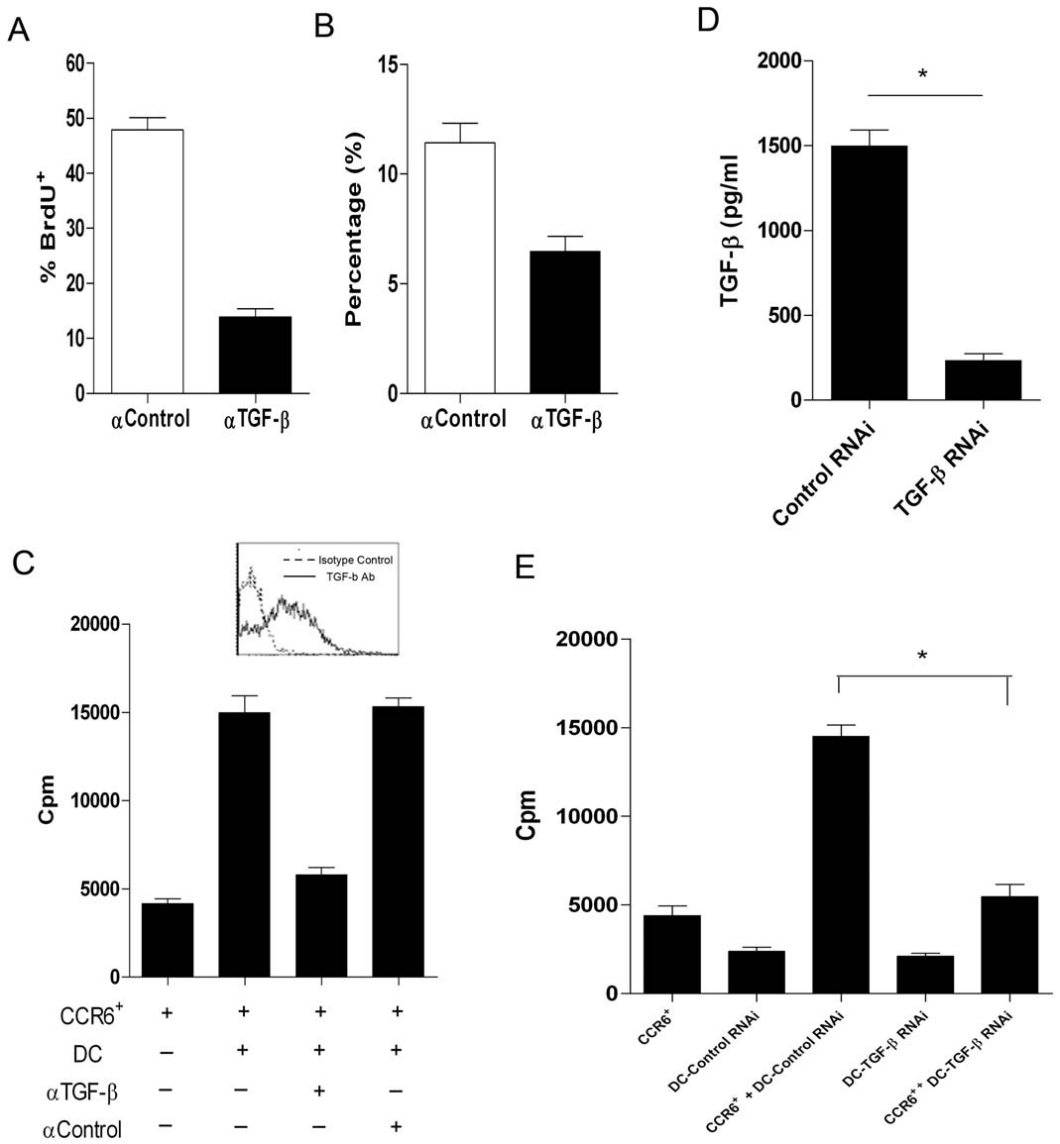
DCs triggerred the proliferation of CCR6^+^Treg in a TGF-β dependent manner. The 4T1-bearing mice were treated with 2 mg BrdU i.p. every other day up to a cumulative dose of 8 mg BrdU. These mice also were simultaneously intratumorally injected 0.1-ml aliquots of Anti-TGF-β antibody (50 ug) (▪) (*n* = 6) or rat IgG2a isotype control (□) (*n* = 6) every other day. 8 hrs after last injection of BrdU, The TILs were collected. The proliferation (**A**) and proportion (**B**) of CCR6^+^Tregs in TILs were analyzed by FACS and calculated. (**C**) Proliferation of CCR6^+^Tregs after culturing with DCs at a ratio of 1∶2 in the presence of anti-TGF-β or isotype control antibody (10 ug/ml) for 3 days. (**D**) CD11c^+^ DCs were purified from TILs by MACS. 1×10^5^ DCs was cultured and transfected with TGF-β RNAi or Scramble control. 72 hrs later, the supernatant concentration of TGF-β were determined by ELSA assay. (**E**) 1×10^5^ CCR6^+^ Tregs were cocultured with DCs transfected with TGF-β RNAi or scramble control at a ratio of 1∶2. 72 hrs later, the proliferation of CCR6^+^Tregs was determined. * p<0.05.

Previous finding demonstrated TGF-β was critical for Treg proliferation in vivo [18], [19]. Hence, anti-TGF-β antibody was administered to mice before analyzing the BrdU^+^CCR6^+^Tregs. As shown in fig. 4a, anti-TGF-β antibody treatment significantly reduced the proliferation and frequency of CCR6^+^Tregs in tumor mass, suggesting TGF-β was critical for the in situ CCR6^+^Tregs proliferation. Then, the expression level of TGF-β in tumor-resident DCs was analyzed. As shown in fig. 4d, tumor-resident DCs expressed high level of TGF-β. anti-TGF-β treatment also abrogated nearly 65% in vitro proliferation of CCR6^+^Tregs triggered by DCs (Fig. 4c, p<0.05). To exclude the influence of CCR6^+^Tregs derived TGF-β on the effects of DCs, we transfected TGF-β RNAi into DCs. As shown in fig. 4d, TGF-β RNAi could significantly reduce the TGF-β secretion of DCs. Importantly, the proliferation of CCR6^+^Tregs in TGF-β-RNAi transfected DCs group decreased significantly compared with control group (Fig. 4e, p<0.05). These data indicated that tumor-derived CD11c^+^DCs triggered the proliferation of CCR6^+^Treg in a TGF-β dependent manner.

### In situ expansion of CCR6^+^Tregs was crucial for their enrichment and suppression in vivo

**Figure 5:**
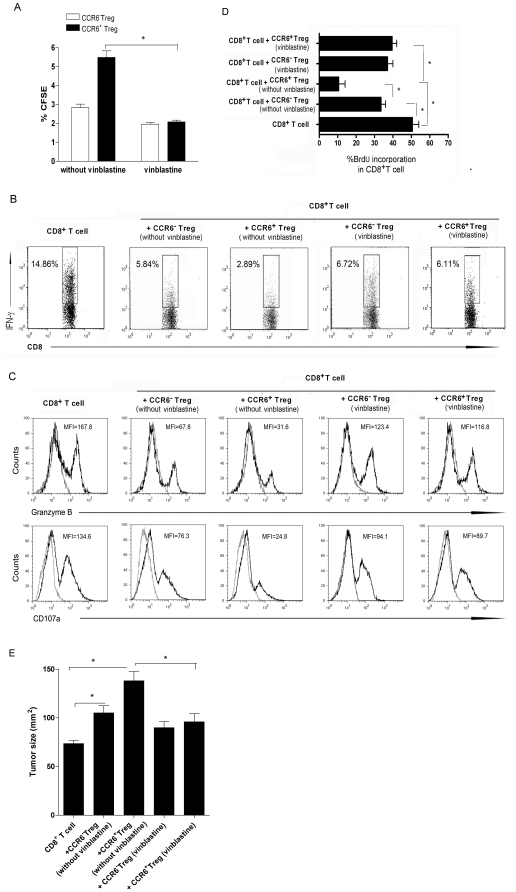
The proliferation of CCR6^**+**^Tregs in situ was crucial for their suppressive function in vivo. Balb/c mice were immunized with inactivated 4T1 tumor cells 3 times, with 1-week-interval. Then CD8^+^ T cells were sorted from splenocytes and re-stimulated with inactivated 4T1 tumor cells for 24 h *in vitro*. CCR6^+^Tregs and CCR6^−^Tregs were purified from TILs and treated with or with vinblastine (50 ng/ml) for 12 hrs. Then CD8^+^T cells were transferred with CCR6^+^Treg or CCR6^−^Tregs pretreated with or without vinblastine at a ratio of 2∶1 into syngeneic 4T1-bearing nude mice (n = 8). 10 days later, the proportion of CCR6^+^Tregs or CCR6^−^Tregs in TILs of 4T1 bearing nude mice was analyzed by FACS and calculated (**A**). CD8^+^T cells in tumor mass also were obtained. The percentage of IFN-γ secreting CD8^+^ T cells (**B**), as well as the expression of CD107a and Granzyme B on CD8^+^T cells (**C**), were analyzed by FACS. Symbols were representative of three independent experiments. Medians were indicated. (**D**) And the proliferation of CD8^+^T cells were also obtained by BrdU incorporation assay and calculated. (**E**) 21 day later, the tumor size in each group also was obtained.* p<0.05.

Our recent work indicated that adoptive transfer of CCR6^+^Tregs more effectively suppressed the anti-tumor CD8^+^T cells than their CCR6^−^ counterparts did [20]. Then, whether in situ prior expansion of CCR6^+^Tregs might be responsible for their enrichment and subsequently suppressive effects in vivo was investigated. As shown in fig. 5a,b, CCR6^+^ or CCR6^−^Tregs were sorted from TILs and pretreated with or without vinblastine. Then cells were transferred into 4T1 bearing syngeneic nude mice with 4T1 specific CD8^+^T cells at a ratio of 1∶2 respectively. As shown in fig. 5a, vinblastine pre-treatment obviously decreased CCR6^+^Treg frequency in tumor mass from 5.43% to 2.13% (p<0.05). Correspondingly, IFN-γ production of CD8^+^T cells in CCR6^+^ Tregs co-transferred mice was 2.89%, while it increased dramatically to 6.11% when the co-transferred CCR6^+^Tregs were pre-treated with vinblastine (Fig. 5b, p<0.05). Similar results were obtained on the proliferation, as well as expression of CD107a and Granzyme B, of CD8^+^T cells (Fig. 5c,d, p<0.05). Finally, the size of tumor mass in CCR6^+^Tregs co-transferred mice was significantly elevated compared with that in CD8^+^T cells transferred group and CCR6^−^Tregs co-transferred group, while it decreased obviously in vinblastine pre-treated CCR6^+^ Tregs co-transferred group(Fig. 5e, *p*<0.05).

In addition, vinblastine pretreatment also limitedly reduced the percentage of CCR6^−^Tregs in tumor mass (Fig. 5a, p>0.05) and led to slightly elevated IFN-γ production and proliferation, as well as the expression of CD107a and Granzyme B, of CD8^+^T cells (Fig. 5b,c,d, p>0.05). Altogether, all our data suggested that in situ dominant proliferation capacity was critical for the enrichment and suppressive effect of CCR6^+^Tregs *in vivo*.

## Discussion

In the present study, we showed that CCR6 ^+^Tregs but not CCR6^−^Tregs were enriched in tumor mass during tumor progression in murine breast cancer model. And it was found that predominant in situ proliferation of CCR6^+^Tregs was critical for their local enrichment and subsequently their suppression in tumor immunity. Tumor resident DCs were found to trigger the proliferation of CCR6^+^Tregs in a TGF-β dependent manner.

Enrichment of CD4^+^CD25^+^regulatory T cells in tumor mass was found in various tumor animal model and clinical tumor patients [1], [4]. Our previous study showed that CCR6^+^Treg cells were dominantly enriched in tumor mass and closely related to poor prognosis of breast cancer patients [14]. In this study, we extended our pervious finding by demonstrating that CCR6^+^ Tregs, but not CCR6^−^ Tregs were enriched in tumor mass during progression of breast cancer. CCR6^+^Tregs displayed a phenotypic and functional characteristic of memory-effector T cells, expressing high level of CD44 and low level of CD62L, which is consistent with other work [11].

Previous evidence suggested that chemokine family members, such as CCL17 and CCL22, direct the movement of Tregs in tumor mass [21], [22]. Recent evidence further indicated that conversion of CD4^+^CD25^−^ T cells into Tregs, as well as local proliferation of Tregs also was contributed to local enrichment of Tregs [23], [24]. Notably, we extended others work by demonstrating that CCR6^+^ Tregs, but not CCR6^−^Tregs, predominantly proliferate in tumor mass. Our recent finding suggested that adoptive transfer of CCR6^+^ Tregs could more potently inhibit the function of CD8^+^T cells in vivo than CCR6^−^Tregs did. Here, we further provided direct evidence showed that prior proliferation of CCR6^+^ Tregs was critical for their local enrichment and subsequently contributed to their suppressive effect on anti-tumor CD8^+^T cells in vivo. Then, our data also suggested that the proliferation of regulatory T cells, as well as conversion of non-regulatory T cells into Treg cells and migration of Treg, was crucial for their enrichment and suppressive capacity in tumor mass.

CD11c^+^ DCs were found by our previously finding to be accumulated in tumor sites during tumor progression [25]. Here, we further found that tumor-resident DCs significantly triggered the proliferation of CCR6^+^T cells in a TGF-β dependent manner. Similar work also reported immature DCs-induced TGF-β-dependent Treg proliferation [26], [27]. Meanwhile, our data found that DLN derived DCs had little effect on the proliferation of CCR6^+^Tregs suggesting that distinct DC subsets have heterogeneous effects on Tregs which awaits further research works.

To the discrepancies on the proliferation of CCR6^+^Tregs and CCR6^−^Tregs in vivo, we presumed that the prior proliferation of CCR6^+^Tregs was dominantly dependent on two factors. On the one hand, memory T cells are more readily activated than naive cells, as evidenced by their ability to respond to lower doses of Ag, by their lesser dependence on co-stimulatory receptor-ligand interactions, and by their enhanced responses to stimulation with TCR antibody [28], [29]. Our present data showed that CCR6^+^Tregs displayed effector-memory T cells phenotype. Rivino et al reported that CCR6^+^ memory T cells secreted cytokine efficiently in response to suboptimal T cell receptor (TCR) stimulation with anti-CD3 antibodies [30]. Kleinewietfeld et al further reported that CCR6^+^Tregs showed more sensitivity to TCR antibody [11]. On the other hand, the functional character of tumor-resident APC might also be critical for the different proliferation of CCR6^+^Tregs and CCR6^−^Tregs in vivo. It is demonstrated that tumor-resident DCs expressed low level of MHC Class-II molecules and co-stimulatory molecules, displayed immature phenotype [26], [31], [32]. Moreover, our present data also showed that tumor-resisent DCs could significantly promote the proliferation of CCR6^+^Tregs but not CCR6^−^Tregs. Therefore, the different proliferation of CCR6^+^Tregs and CCR6^−^Tregs in tumor mass might be partly attributed to their different sensitivity following interaction with intra-tumoral DCs. However, the exact mechanism remains to be elucidated in successive work.

In all, this research represents as the first report to elucidate the mechanism of the selective enrichment of CCR6^+^Tregs but not CCR6^−^Tregs in tumor mass. And tumor-resident DC secreting TGF-β seems to be crucial for the in situ expansion and suppressive effect of CCR6^+^Tregs *in vivo*. This study might throw a new insight on the understanding of orientation and function-sustaining mechanism of unique regulatory T cell subset in the setting of breast tumor and provide helpful thoughts for the designing of Treg-based immunotherapy for tumor in the future.

## Materials and methods

### Animals

Female Balb/c mice and Balb/c nude mice 5–6 weeks of age were purchased from the Center of Experimental Animal, Fudan University (Shanghai, China). All animals were housed in the pathogen free mouse colony at our institution and all animal experiments were performed according to the guidelines for the Care and Use of Laboratory Animals (Ministry of Health, PR China, 1998) and all the experimental procedure was approved by the “the ethical guidelines of Shanghai Medical Laboratory Animal Care and Use Committee” (permit number:2007013).

### Cell lines

4T1 cell line, a Balb/c spontaneous metastatic mammary carcinoma, were cultured at 37°C under 5% CO_2_ in completed RPMI 1640 (GIBICO, Grand land, NY, USA) medium containing 10% heat-inactivated fetal bovine serum and supplemented with 2 mM glutamine, 100 IU/ml penicillin, and 100 ug/ml streptomycin sulfate.

### Nude mice tumor model and co-transfer experiment

Syngeneic female nude mice were injected subcutaneously with 0.2 ml of a single-cell suspension containing 2×10^5^ 4T1 adenocarcinoma cells in the right anterior mammary fat pad region. 7 days later, 4T1 specific CD8^+^T cells were transferred with or without CCR6^+^Tregs and CCR6^−^Tregs at a ratio of 2∶1 through tail vein respectively. 21 days later, the size of tumor mass in each group was obtained as previous described [3]. To obtain 4T1 specific CD8^+^T cells, Balb/c mice were immunized with inactivated 4T1 tumor cells 3 times, with 1-week-interval. Then CD8^+^ T cells were sorted from splenocytes and re-stimulated with inactivated 4T1 tumor cells for 24 h *in vitro*.

### Isolation of TILs

The lymphocytes were harvested from 4T1 tumors by a discontinuous density gradient method. Briefly, 4T1 tumors were removed aseptically and minced with scissors into 1–2 mm^3^ pieces. The minced tumors were then stirred in 40 ml complete RPMI 1640 containing 40 mg collagenase, type IV(Sigma), 4 mg deoxyribonuclease (Sigma) and 100 U hyaluronidase (Sigma) for 3 h at room temperature. The tumor cell suspension was filtered through a nylon-mesh screen with pores of 50 µm to remove cell clumps, and the filtrate was then centrifuged (250 *g*, 10 min). The cell pellet was washed twice with serum-free RPMI 1640 and resuspended in complete RPMI 1640. A 4-ml aliquot of cell suspension of disaggregated tumor was placed on top of the gradient formed by overlapping a cushion of 100% Ficoll-Paque (Pharmacia Fine Chemicals, Piscataway, NJ) with an equal volume of 75% Ficoll-Paque in RPMI 1640. Gradients (14 ml) were centrifuged at 800 *g* for 30 min at room temperature. The distinct band formed at the interface between 75% and 100% Ficoll-Paque was collected and washed three times in fresh medium.

### Flow cytometry

Flow cytometry was performed on a FACS Calibur (BD Biosciences) with CellQuest Pro software using directly conjugated mAbs against the following markers: CD3-FITC, CD4-PerCP, CD25-allophycocyanin, CCR6-PE with corresponding isotype-matched controls (either BD Biosciences or eBioscience Systems). Foxp3 staining was conducted using the mouse Regulatory T cell staining kit (eBioscience) and run according to the manufacturer's protocol. To determine the percentage of CCR6^+^ Tregs and CCR6^−^Tregs, lymphocytes were gated by plotting forward vs side scatter followed by gating on CD4^+^CD25^high^ T cells, and these cells were then analyzed for Foxp3 expression and CCR6 expression.

To determine the expression level of CD107a on CD8^+^ T cells, after staining of surface markers (CD8), cells were washed twice with PBS. Antibodies to CD107a and corresponding isotype controls were obtained from BD Biosciences. Cells were stained with antibody against CD107a (1∶100) at 25°C for 20 min and washed before analysis.

### Suppression assays

To test CCR6^+^ Treg cells suppressive activity, 5×10^4^ CD4^+^CD25^−^ cells were treated with 2 µg/ml anti-CD3(eBioscience) and anti-CD28 (eBioscience) for 12 hrs as effector cells, then incubated with or without CCR6^+^ Tregs at a ratios of 2∶1 for 72 hrs in complete medium containing RPMI 1640 (Sigma, St. Louis, MO) supplemented with 5% FCS. ^[3^H^]^thymidine (0.5 µCi/well) was added for the last 18 hours of culture.

### BrdU labeling

The 4T1 bearing Balb/c mice and syngeneic nude mice were treated with 2 mg BrdU(5-bromo-2-deoxyuridine; Sigma) i.p. every other day up to a cumulative dose of 8 mg BrdU as indicated. Eight hours after the last BrdU injection, TILs recovered from the tumor mass were analyzed by flow cytometry for their incorporation of BrdU. In brief, TILs were stained with antibodies for cell surface markers and fixed and permeabilized using Cytofix/Cytoperm and Perm/Wash buffer (BD Biosciences) according to the manufacturer's instructions. Cells were incubated at 24°C for 30 min in 0.15 M NaCl, 4.2 mM MgCl_2_, 10 mM HCl, pH 5 in the presence of 2 U DNase I (Invitrogen), followed by staining with αBrdU-FITC (eBioscience) for 30 min and were finally analyzed by FACS.

### Design of artificial siRNAs and transfection

We designed the TGF-β targeting sequence (sense, 5′-GACCAUCGACAU- -GGAGCUG-3′). The nonspecific control siRNA duplexes were purchased from Dhamacon with the same GC content as TGF-β siRNAs (52.4%, D001810-02). These siRNA gene double-strands were ligated with pSilencer2.0 U6 RNAi Expression Vector (Ambion). Then, the vector with TGF-β–siRNA or control siRNA plasmids were transiently transfected into the CD11^+^DC cells using Lipofectamine-2000 (Invitrogen) according to the manufacturer's instruction.

### Chemotaxis assay

Chemotaxis assays were performed using 24-well Transwell chemotaxis plates (5-µm pore size; Corning Costar) as described previously [15]. Cell culture supernatants from 4T1 tumor cell lines or various concentrations of recombinant murine CCL17, CCL20, or CCL22 (100 ng/mL; R&D Systems, Minneapolis, MN) in RPMI 1640 medium were added to the lower chamber of the Transwell plates. CCR6^+^Tregs or CCR6^−^Tregs (1×10^5^ cells) were transferred into upper chambers. After 150 min at 37°C, chemotaxis was quantified by detecting the numbers of cells that migrated into the lower chamber. The chemotaxis index was calculated by dividing the numbers of cells migrated in response to test supernatants or recombinant human chemokines by the numbers of cells migrated in response to medium alone.

To obtain culture supernatant, 4T1 cell lines (0.5×10^6^/ml) were cultured for 48 hrs. The culture supernatants then were collected and used in chemotaxis assays. For Ab-blocking assays, various concentrations of neutralizing anti-CCL22 (500 ng/mL), anti-CCL17(500 ng/mL), and anti-CCL20 (500 ng/mL) (R&D Systems) Abs were added to supernatants and incubated at 37°C for 30 min before performing chemotaxis assays.

### Enzyme-linked immunosorbent assays

Tumor cell lines (0.5×10^6^/ml) were cultured for 48 hrs. The culture supernatants were collected and performed using Quantikine Immunoassay kits specific for murine CCL17, CCL22 and CCL20, according to the manufacturer's instructions (all from R&D Systems).

### Intracellular staining for IFN-γ and Granzyme B

TILs were isolated from 4T1 bearing syngeneic nude mice transferred with CD8^+^T cells, CD8^+^T cells plus CCR6^+^Treg and CD8^+^T cells plus CCR6^−^Treg respectively as indicated time point. After staining of surface markers (CD8), cells were fixed and permeabilized using Cytofix/Cytoperm and Perm/Wash buffer from BD Biosciences according to the manufacturer's instructions. Antibodies to IFN-γ or Granzyme B and corresponding isotype controls were obtained from BD Biosciences. Cells were stained with antibody against IFN-γ (1∶100) or antiboy against Granzyme B (1∶100) at 25°C for 20 min and washed twice in Perm/Wash before analysis.

### CFSE labeling

Cells were purified from 4T1 bearing mice by FACS sorting and labeled with 5-carboxy-fluorescein diacetate succinimidyl ester (CFSE; Molecular Probes, Leiden, The Netherlands) as previously described [3]. 2×10^6^ CFSE-labeled cells were intratumorally injected into 4T1 bearing syngeneic mice. 10 days later, single-cell suspensions of tumor infiltrating lymphocytes were prepared and the proliferation of cells were analyzed by FACS.

### Statistical analyses

Statistical analyses of the data were performed with the aid of analysis programs in SPSS12.0 software. Statistical evaluation was performed using one way analysis of variance (ANOVA; *p*<0.05) or t test using the program PRISM 4.0 (GraphPad Software Inc., San Diego, CA, USA).

## Supporting Information

Figure S1 **The migration of CCR6^+^ Treg cells and CCR6^−^Treg cells in response to CCL17/CCL22 and CCL20.** The migration of CCR6^+^Tregs and CCR6^−^Tregs in response to CCL17/CCL22 (100 ng/ml) or CCL20 (100 ng/ml) were perfomed by transwell migration assays respectively as described in *Material and Methods*. One representative data of three independent experiments was shown. *p<0.05.
(DOC)

Figure S2 **The supernatant concentration of CCL17, CCL20 and CCL22.** 4T1 tumor cell lines (5×10^5^/ml) were cultured for 48 hrs. The culture supernatants were collected and the concentration of CCL17, CCL20 and CCL22 were determined by ELISA assay and calculated. One representative data of three independent experiments was shown.
(DOC)

Figure S3 **CCR6^+^ Treg cells and CCR6^−^Treg cells migrate in response to 4T1 supernatants.** 4T1 tumor cell lines (5×10^5^/ml) were cultured for 48 hrs. The culture supernatants were collected. The migration of CCR6^+^Tregs or CCR6^−^Tregs in response to supernatants were perfomed by transwell migration assays as described in *Material and Methods*. After preincubation for 30 minutes at 4°C with anti-CCL17, anti-CCL20 and anti-CCL22 mAbs (500 ng/mL), CCR6^+^Tregs and CCR6^−^ Tregs migration were also tested by transwell migration assays respectively. One representative data of three independent experiments was shown. *p<0.05.
(DOC)
