# Supplementary material for: In Situ Prior Proliferation of CD4+ CCR6+ Regulatory T Cells Facilitated by TGF-β Secreting DCs Is Crucial for Their Enrichment and Suppression in Tumor Immunity
Source: PLoS One. 2011 May 31;6(5):e20282. doi: 10.1371/journal.pone.0020282 (PMC3105045; doi:10.1371/journal.pone.0020282)
Supplement: Figure S1 — The migration of CCR6+ Treg cells and CCR6−Treg cells in response to CCL17/CCL22 and CCL20. The migration of CCR6+Tregs and CCR6−Tregs in response to CCL17/CCL22 (100 ng/ml) or CCL20 (100 ng/ml) were perfomed by transwell migration assays respectively as described in Material and Methods. One representative data of three independent experiments was shown. *p<0.05. (DOC) [file pone.0020282.s001.doc]

Supplementary Fig 1


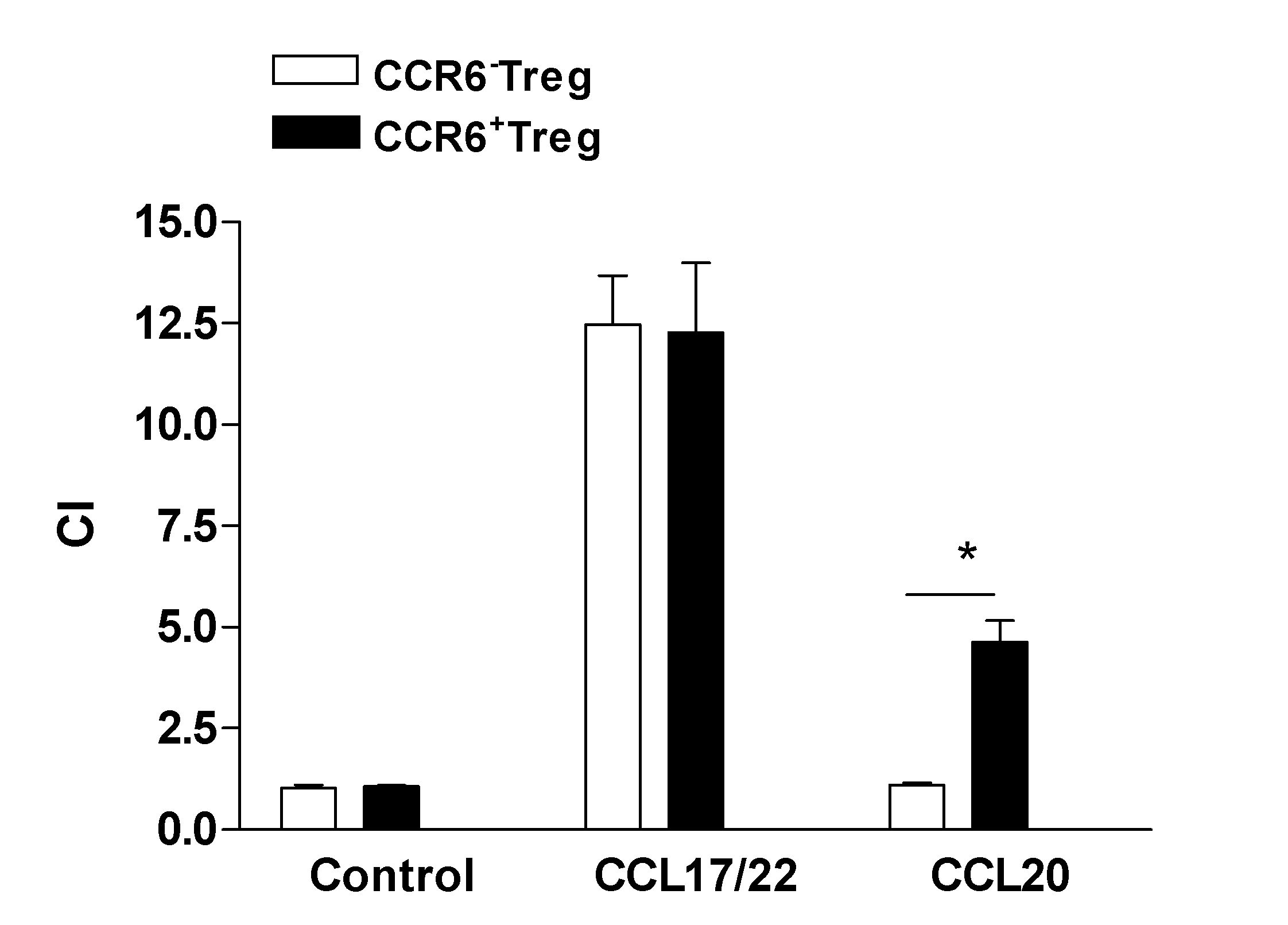


Fig 1. The migration of CCR6^+^ Treg cells and CCR6^-^Treg cells in response to CCL17/CCL22 and CCL20.

The migration of CCR6^+^Tregs and CCR6^-^Tregs in response to CCL17/CCL22 (100ng/ml) or CCL20 (100ng/ml) were perfomed by transwell migration assays respectively as described in *Material and Methods.* One representative data of three independent experiments was shown. **p*<0.05.
