# Supplementary material for: In Situ Prior Proliferation of CD4+ CCR6+ Regulatory T Cells Facilitated by TGF-β Secreting DCs Is Crucial for Their Enrichment and Suppression in Tumor Immunity
Source: PLoS One. 2011 May 31;6(5):e20282. doi: 10.1371/journal.pone.0020282 (PMC3105045; doi:10.1371/journal.pone.0020282)
Supplement: Figure S2 — The supernatant concentration of CCL17, CCL20 and CCL22. 4T1 tumor cell lines (5×10^5^/ml) were cultured for 48 hrs. The culture supernatants were collected and the concentration of CCL17, CCL20 and CCL22 were determined by ELISA assay and calculated. One representative data of three independent experiments was shown. (DOC) [file pone.0020282.s002.doc]

Supplementary Fig 2


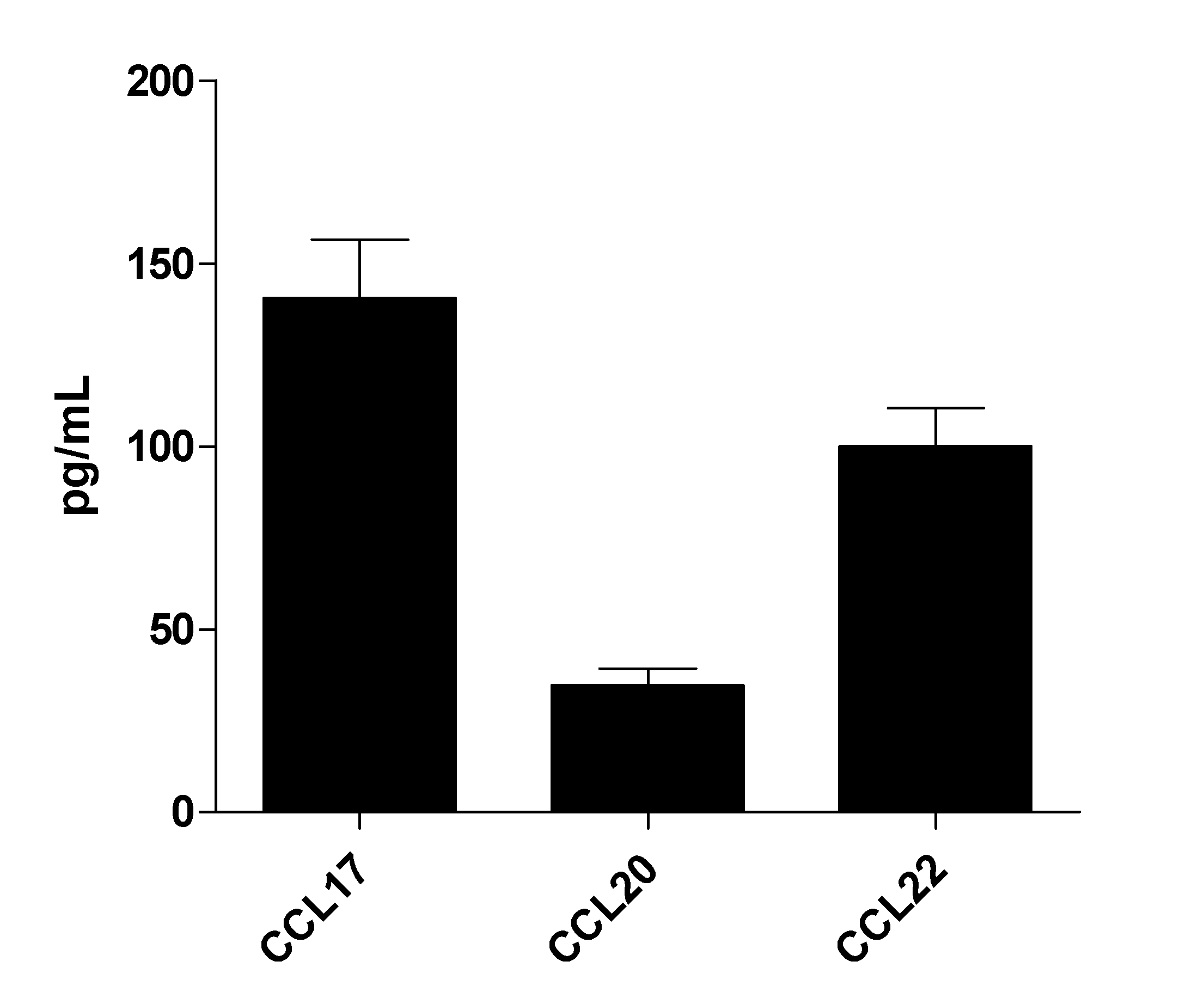


**Fig 2. The supernatant concentration of CCL17, CCL20 and CCL22.**

4T1 tumor cell lines (5× 10^5^/ml) were cultured for 48 hrs. The culture supernatants were collected and the concentration of CCL17, CCL20 and CCL22 were determined by ELISA assay and calculated. One representative data of three independent experiments was shown.
