# Supplementary material for: In Situ Prior Proliferation of CD4+ CCR6+ Regulatory T Cells Facilitated by TGF-β Secreting DCs Is Crucial for Their Enrichment and Suppression in Tumor Immunity
Source: PLoS One. 2011 May 31;6(5):e20282. doi: 10.1371/journal.pone.0020282 (PMC3105045; doi:10.1371/journal.pone.0020282)
Supplement: Figure S3 — CCR6+ Treg cells and CCR6−Treg cells migrate in response to 4T1 supernatants. 4T1 tumor cell lines (5×10^5^/ml) were cultured for 48 hrs. The culture supernatants were collected. The migration of CCR6+Tregs or CCR6−Tregs in response to supernatants were perfomed by transwell migration assays as described in Material and Methods. After preincubation for 30 minutes at 4°C with anti-CCL17, anti-CCL20 and anti-CCL22 mAbs (500 ng/mL), CCR6+Tregs and CCR6− Tregs migration were also tested by transwell migration assays respectively. One representative data of three independent experiments was shown. *p<0.05. (DOC) [file pone.0020282.s003.doc]

Supplementary Fig 3


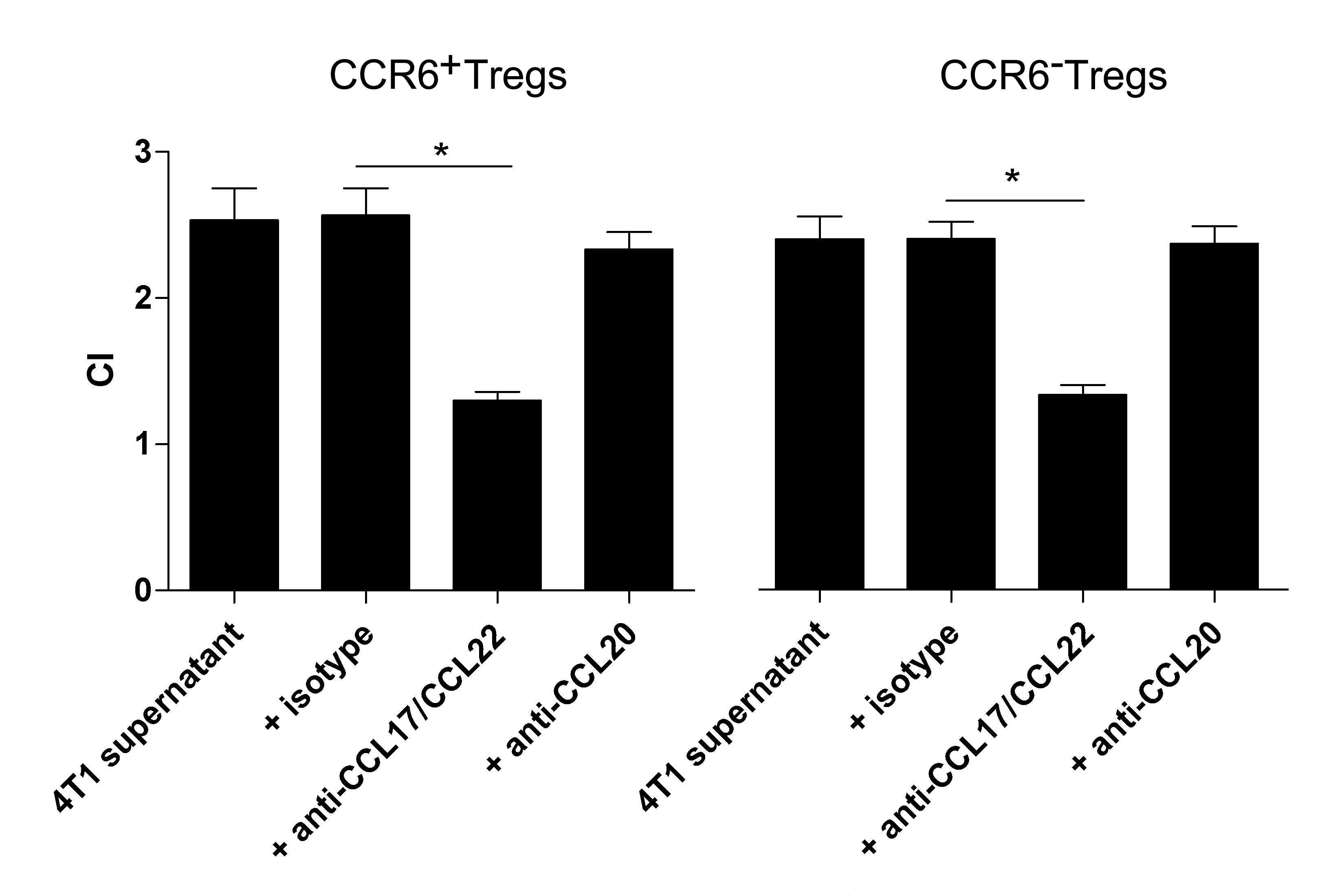


Fig 3. CCR6^+^ Treg cells and CCR6^-^Treg cells migrate in response to 4T1 supernatants.

4T1 tumor cell lines (5× 10^5^/ml) were cultured for 48 hrs. The culture supernatants were collected. The migration of CCR6^+^Tregs or CCR6^-^Tregs in response to supernatants were perfomed by transwell migration assays as described in *Material and Methods.* After preincubation for 30 minutes at 4°C with anti-CCL17, anti-CCL20 and anti-CCL22 mAbs (500 ng/mL), CCR6^+^Tregs and CCR6^-^ Tregs migration were also tested by transwell migration assays respectively. One representative data of three independent experiments was shown. **p*<0.05.
